# Value-Added Compound Recovery from Invasive Forest for Biofunctional Applications: *Eucalyptus* Species as a Case Study

**DOI:** 10.3390/molecules25184227

**Published:** 2020-09-15

**Authors:** Patricia Gullón, Beatriz Gullón, Gonzalo Astray, Paulo E. S. Munekata, Mirian Pateiro, José Manuel Lorenzo

**Affiliations:** 1 Nutrition and Bromatology Group, Department of Analytical and Food Chemistry, Faculty of Food Science and Technology, University of Vigo, Ourense Campus, 32004 Ourense, Spain; pgullon@uvigo.es; 2 Department of Chemical Engineering, Faculty of Science, University of Vigo (Campus Ourense), As Lagoas, 32004 Ourense, Spain; bgullon@uvigo.es; 3 Department of Physical Chemistry, Faculty Science, Faculty of Science, University of Vigo (Campus Ourense), As Lagoas, 32004 Ourense, Spain; gastray@uvigo.es; 4 CITACA, Agri-Food Research and Transfer Cluster, Campus Auga, University of Vigo, 32004 Ourense, Spain; 5 Centro Tecnológico de la Carne de Galicia, Rúa Galicia No 4, Parque Tecnológico de Galicia, San Cibrao das Viñas, 32900 Ourense, Spain; paulosichetti@ceteca.net (P.E.S.M.); mirianpateiro@ceteca.net (M.P.); 6 Área de Tecnología de los Alimentos, Facultad de Ciencias de Ourense, Universidad de Vigo, 32004 Ourense, Spain

**Keywords:** biomass, innovative extraction technologies, polyphenols, biological properties

## Abstract

From ancient times, the medicinal properties of the different *Eucalyptus* species are well known. In fact, plants from this family have been used in folk medicine as antiseptics, and to treat different ailments of the upper respiratory tract such as sinus congestion, common cold, or influenza. Moreover, other biological activities were described for *Eucalyptus* species such as antioxidant and antimicrobial properties. In the last few decades, numerous investigations revealed that the compounds responsible for these properties are secondary metabolites that belonging to the group of phenolic compounds and are present in different parts of the plants such as leaves, bark, wood, fruits, and stumps. The increasing demand for natural compounds that can substitute synthetic antioxidants and the increase in resistance to traditional antibiotics have boosted the intense search for renewable natural sources containing substances with such bioactivities, as well as greener extraction technologies and avant-garde analytical methods for the identification of the target molecules. The literature data used in this paper were collected via Scopus (2001–2020) using the following search terms: *Eucalyptus*, extraction methods, phenolic compounds, and biological activities. This review collects the main studies related to the recovery of value-added compounds from different *Eucalyptus* species, as well as their biofunctional applications.

## 1. Introduction

In the last few years, there has been an increase in social concern regarding health issues, as well as a trend toward the use of natural compounds, leading to great efforts devoted to the search of new biosources rich in bioactive molecules with beneficial properties for human health and wellbeing [1]. Therefore, the presence of natural products in the pharmaceutical, food processing, and supplement production industries is growing [2].

In this context, forest biomass produces secondary metabolites that are bioactive molecules with antibacterial properties and low toxicity, which are considered potential candidates for developing a new generation of antimicrobial agents [3,4]. Since ancient times, several parts of forest biomass have been used to treat or prevent different diseases. This popular use has encouraged an intense investigation to identify secondary biomass metabolites as a source of medicinal agents [5].

In this sense, in the last few years, the growing interest in replacing synthetic antioxidants has fostered research in renewable secondary biomass metabolites to obtain natural and low-cost antioxidants that can substitute synthetic preservatives such as butylated hydroxyanisole (BHA), butylated hydroxytoluene (BHT), and/or propyl gallate and terbutyl hydroquinone (TBHQ) [4,6,7,8], showing that they present toxic, carcinogenic, and harmful effects on human health. These natural antioxidant compounds have applications in the field of foods by preventing lipid peroxidation, and they can also be used as protective agents against oxidative damage and food-borne spoilage microorganisms [7,9,10,11,12]. Moreover, natural antioxidants can be used in topical pharmaceutical and cosmetic compositions [4,13].

The genus *Eucalyptus* (Myrtaceae family) is indigenous to Australia and encompasses more than 900 species and subspecies [14]. It is an invasive species, which is highly inflammable and responsible for forest fire. Its wood is used in pulp and paper manufacture due to its fast-growing and short rotation periods and excellent properties of pulping and bleaching [15]. Moreover, different species of *Eucalyptus* are used in folk medicine as antiseptics due to their antimicrobial properties, and to treat respiratory infections (common cold, influenza, and/or sinus congestion) [16]. Furthermore, the essential oils from this genus are well known to display several bioactivities such as antibacterial, antifungal, analgesic, and anti-inflammatory properties [14,17].

In recent years, the different parts of the trees belonging to the Myrtaceae family such as bark, leaves, branches, fruits, and knots have attracted much attention as promising sources of high-value phytochemicals [15]. In particular, *Eucalyptus* species are excellent sources of bioactive terpenoids, tannins, flavonoids, and phloroglucinol derivatives [18].

An essential aspect in the field of extraction of biomolecules from natural sources is the selection of the more suitable technology, as well as of the solvent, to guarantee a high yield and to maintain the bioactivities of the recovered extract such that, when it is incorporated in a food matrix, it maintains its functionality. In the recent past, the recovery of phytochemicals from natural sources was based on conventional methods that use organic solvents and their aqueous mixtures. Currently, it is known that these methods present drawbacks with negative consequences for the bioactivities of the recovered phytochemicals. Consequently, in recent years, there has been an increase in the development of eco-friendly and sustainable avant-garde technologies combined with smart solvents to preserve the functionality of the extracted compounds. In the last decade, ultrasound-assisted extraction (UAE), microwave-assisted extraction (MAE), supercritical fluid extraction (SFE), and subcritical water extraction (SWE) were outlined as safe and effective technologies in the field of recovery of biomolecules from forest biomass [19,20,21,22,23].

This review collects the studies related to the recovery of phytochemicals from different *Eucalyptus* species. Aspects such as the main phenols identified in extracts recovered from different parts of the plant belonging to the *Eucalyptus* species, the different extraction technologies from conventional to innovative employed, and the biofunctional properties assayed until date of the recovered extracts are addressed.

## 2. Phenolic Compounds from *Eucalyptus* Species

Phenolic compounds are secondary plant metabolites [24] that are present in the plant kingdom [24,25] and are extracted from different parts of plants [26,27,28,29,30].

According to the World Health Organization (WHO), *Eucalyptus* is reported to have different medicinal uses including as an expectorant or to treat asthma, influenza, diarrhea, and fungal infections, among others [2,31]. These compounds can be located in some plants at a high level of content and, due to this fact, these plants have an important role as natural antioxidants [32].

*Eucalyptus* species contain different important amounts of phenolic compounds which confer the antioxidant activity of their extracts [31,33]. Different phenolic compounds such as ellagic acid, rutin, and quercetin, among others have been isolated from different *Eucalyptus* extracts [31,33,34], and, according to a research carried out by Santos et al. [15], *Eucalyptus* species (*E. grandis*, *E. urograndis* (*E. grandis* × *E. urophylla*), and *E. maidenii*) present high potential as good sources of biologically active phenolics.

Almeida et al. [31] studied the phenolic profile of *E. globulus* leaves using high-performance liquid chromatography (HPLC) with a reversed-phase Spherisorb ODS2 column and a C18 guard column. According to the authors, four phenolic compounds were identified and quantified (mg/g of lyophilized extract) in the leaf extracts: flavonoids (rutin 4.4 and quercitrin 2.4) and phenolic acids (chlorogenic acid 4.5 and ellagic acid 2.7). A similar study was carried out by Santos-Ferreira et al. [35] who studied the phenolic compounds in extracts from dried *E. globulus* leaves. Phenolic compounds were extracted upon mixing the dried leaves with different solvents: chloroform, ethanol, methanol, or methanol/water (70:30, *v*/*v*) and then purified using solid-phase extraction (SPE) [35]. The HPLC–MS/MS analysis showed the existence of chlorogenic acid, rutin, and quercetin 3-glucuronide in the purified methanol/water extract, as well as ellagic acid derivatives (dried *Eucalyptus* leaves) [35].

The phenolic composition of other *Eucalyptus* species has also been determined. For example, Nasr et al. [36] carried out extractions for five different species of *Eucalyptus* (*E. maidenii*, *E. robusta*, *E. citriodora*, *E. tereticornis*, and *E. camaldulensis*) to compare their content in some primary and secondary metabolites. The authors identified different phenolic compounds, varying their abundance in *Eucalyptus* leaves, with eight individual phenolics (hydroquinone, hesperitin, pyrogallol, resorcinol, protocatechuic acid, naringenin, chlorogenic acid, and catechin) highlighted in *E. camaldulensis* leaves and gallic highlighted in *E. tereticornis* leaves [36]. Another interesting research was carried out by Akamura et al. [34] who tried to elucidate the constituents of a commercial *Eucalyptus* leaf extract using HPLC and GC/MS. Different phenolic compounds such as gallic acid and ellagic acid were detected and quantified (1.68 mg/g and 1.32 mg/g of *Eucalyptus* product weight, respectively). Moreover, they also found different mono- and sesquiterpenes, such as 1,8-cineole, α-terpineol acetate, aromandendrene, globulol, and sesquiterpene alcohol.

*E. globulus* leaves were also used by Dezsi et al. [37] to determine their phenolic profile. The ethanolic extracts were analyzed by HPLC/MS, and significant amounts of phenolic compounds were found. The major flavonoid compound was hyperoside (666.4 μg/g dry weight (dw) plant material), followed by quercitrin (287.8 μg/g dw plant material) and myricetin (92.3 μg/g dw plant material). Other polyphenols (rutin, isoquercitrin, luteoline, and apigenine, among others) were identified in minor amounts [37].

The extraction of phenolic compounds is also possible from other parts of *Eucalyptus*, not only from the leaves. In this case, Nasr et al. [38] examined the phenolic profile of four organs from *E. camaldulensis* using GC/MS, namely, leaves, buds, empty capsules, and seeds. The GC/MS analysis confirmed that the presence of some phenolic compounds varied depending on the part of the plant under investigation. The most abundant phenolic compounds were gallic acid, catechin, and tricetin with relative abundances of 1898.8%, 1360.8%, and 1323.8%, which were mainly present in the plant seed compared to the other studied parts. According to the data provided by the authors, generally, the seed showed the highest content for some of the detected compounds including hydroquinone, pyrogallol, and taxifolin, among others, while the leaf presented the greatest abundance of hesperitin, resorcinol, and chlorogenic acid, the bud presented the highest neohesperidin content, and the plant capsule presented the second highest contents of tricetin, gallic acid, and catechin.

Vázquez et al. [39] used matrix-assisted laser desorption ionization time-of-flight (MALDI-TOF) mass spectrometry and reverse-phase high-performance liquid chromatography/electrospray ionization time-of-flight (RP-HPLC–ESI-TOF) mass spectrometry to identify the different phenolic antioxidants from *E. globulus* bark aqueous extracts. This study confirmed the presence of polygalloylglucoses mixtures (gallotannins), catechin, and epicatechin or ellagic acid, among others.

Santos et al. [15] identified the phenolic profile in bark extracts (methanol/water 50:50) of various species: *E. grandis, E. maidenii*, and *E. urograndis* (*E. grandis × E. urophylla*). Different phenolic compounds were identified for each species (13 for *E. grandis*, 24 for *E. maidenii*, and 12 for *E. urograndis*), with ellagic acid–rhamnoside, dihydroxy-isopropylchromone–hexoside, and dihydroxy-(methylpropyl)isopropylchromone–hexoside referenced for the first time in *Eucalyptus* species by these authors. According to the data provided, the predominant phenolic compounds in *E. grandis* and *E. urograndis* bark were epicatechin and quercetin–glucuronide, while those in *E. maidenii* bark were catechin, chlorogenic acid, and methyl-ellagic acid–pentose [15].

The by-products generated from the *Eucalyptus* wood industry were also studied to identify compounds with antioxidant potential. For instance, Celeiro et al. [40] analyzed three by-products from *Eucalyptus* greenboard manufacture, namely, the water derived from the washing of the wood chips, the condensates, and the concentrate of *Eucalyptus*. Using GC/MS and LC–MS/MS, the authors were able to identify up to 48 and 30 different compounds in the water from the washing of the wood chips and condensates, respectively, while the organic extracts derived from the concentrate of *Eucalyptus* presented up to 72 compounds. Chromatographic analyses highlighted the presence of monoterpenes, sesquiterpenes, polyphenols, and precursors of fragrance synthesis, as well as other biomolecules with antioxidant activity.

Using pyrolysis and gas chromatography coupled to mass spectrometry (Py-GC/MS) analysis, Gullón et al. [41] identified 21 compounds (β-eudesmol, γ-eudesmol, and globulol, among others) from *Eucalyptus* leaf extract. The authors also conducted a Fourier-transform infrared spectroscopy (FTIR) analysis, revealing specific bands that can be attributed to different phenolic compounds such as flavonoids and polyphenols. More recently, related authors carried out a tentative identification of phenolic compounds in different extracts of *E. globulus* leaves using liquid chromatography coupled to trapped ion mobility spectrometry and TOF high-resolution mass spectrometry (UHPLC–TOF MS). The results revealed the possible presence of different bioactive molecules such as sideroxylonal A or B, quercetin 3-*O*-β-d-glucuronide, and ellagic acid– or methylellagic acid–pentoside, among others (*m*/*z* 499.161, 477.067, 300.999, and 447.057, respectively) [42]. Table 1 summarizes some of the most commonly used methods to identify and quantify phenolics from *Eucalyptus* extracts.

## 3. Extraction Procedures of Polyphenols from *Eucalyptus* Species: from Conventional to Innovative Technologies

The efficient recovery of phytochemicals from natural sources is mainly influenced by the method and the conditions used for extraction [27,44,45,46,47,48]. This stage must assure a high extraction performance and preserve the quality of the extracted compounds, in addition to meeting other requirements such as versatility, cost-effectiveness, and ease of operation [49] The most commonly applied method for the recovery and isolation of natural bioactive substances such as polyphenols is inarguably conventional solvent extraction [41,42]. Nevertheless, these processes are relatively inefficient due to them requiring large amounts of hazardous organic solvents, long periods of time, and high extraction temperatures, not to mention the need to remove the solvent to avoid contamination of the extracted compounds [50]. These inconveniences have motivated researchers to look for other more economically feasible and greener technologies for the obtaining of phenolics from several plant matrices [50]. Among these modern technologies, ultrasound-assisted extraction (UAE), microwave-assisted extraction (MAE), supercritical fluid extraction (SFE), and subcritical water extraction (SWE) have been applied to enhance the extraction efficiency and reduce the environmental impact of the conventional processes [2,33,42]. Some studies reported in the literature related to the extraction of phenolics from different species of *Eucalyptus* are presented in Table 2 and discussed in the sections below.

### 3.1. Conventional Technologies

Conventional extraction methods such as Soxhlet, maceration, or hydrodistillation are widely used for the isolation of polyphenols. Several types of solvents such as ethanol, methanol, acetone, diethyl ether, and ethyl acetate in combination with water have been employed for the recovery of these compounds from *Eucalyptus* biomass. It is important to mention that conventional extraction is influenced by different factors (i.e., extraction time, temperature, and concentration of solvent) that must be carefully optimized to achieve high recovery of the target compounds. In this context, Gullón et al. [41] examined the effects of the operating conditions (temperature: 25–50 °C, time: 30–300 min, and percentage of ethanol: 20–80%) on the extraction of compounds with antioxidant capacity from *E. globulus* leaves through response surface methodology (RSM). The results provided by the authors indicated that ethanol percentage had the greatest impact on all the dependent variables analyzed (extraction yield (EY), total phenolic content (TPC), total flavonoid content (TFC), and antioxidant activity determined by DPPH, ABTS, and FRAP). Optimal conditions for extraction were 50 °C, 225 min, and 56% ethanol. Under these conditions, an extraction yield of 32.7%, TPC of 92.7 mg GAE/g dried leaf, TFC of 53.7 mg RE/g dried leaf, and a high antioxidant activity (DPPH, ABTS, and FRAP of 205.4 mg, 363.4, and 185.2 mg TE/g dried leaf, respectively), were achieved [41].

The influence of the type of solvent on the extraction efficiency of phenolic compounds from *E. globulus* biomass was evaluated by several authors. Luis et al. [53] found that of the four solvents tested in stump wood of *E. globulus* (*n*-hexane, ethanol, methanol, and 75% aqueous ethanol), methanol led to the highest extraction yield (12.3%). Regarding the content of phenolic compounds, the three polar solvents yielded similar values; however, the highest flavonoid content was obtained for *n*-hexane extracts. All the extracts, except for those of *n*-hexane, exhibited strong antioxidant activity. In another study, Santos-Ferreira et al. [35] evaluated four solvents, namely, chloroform, ethanol, methanol, and 70% methanol, for the recovery of phenolic compounds from *E. globulus* leaves, and they found that methanol and aqueous methanol were the solvents that led to a higher recovery of phenolics. More recently, Nasr et al. [38] reported that 70% acetone is the most suitable solvent for the extraction of phenolic compounds from *E. camaldulensis* leaves, while the most outstanding antioxidant activity was found in the extracts obtained with 95% acetone.

As previously mentioned, although these solvents are used in industrial processes for the extraction of phenolic compounds from different natural sources, their use has several disadvantages since they cause health risks and environmental problems. To overcome these drawbacks, deep eutectic solvents (DES) were recently proposed as sustainable and safe alternatives to replace common organic solvents in the extraction of phenolic compounds [63]. These solvents are characterized by their excellent biocompatibility, low toxicity, availability, and low cost [19]. These solvents were used for the extraction of specific compounds from some natural sources, such as rutin from the flower buds of *Sophora japonica* and tartary buckwheat hull [64,65]. However, DES applied to *Eucalyptus* biomass was only reported by Gullón et al. [42] who investigated the ability of three DESs formulated with choline chloride (Ch) and ethylene glycol (EG), xylitol (X), and glucose (G) and another mixture containing glucose and citric acid (CA) to extract natural antioxidants from *Eucalyptus* leaves. The authors found that only the blends synthesized with ChEG led to similar TPC and TFC values to those obtained using 56% ethanol (considered as a benchmark); however, the antioxidant capacity of this extract was greatly lower (between 1.1- and 2.6-fold lower compared to the ethanolic extracts).

### 3.2. Innovative Extraction Technologies

In order to improve the extraction performance, reduce the operation time and the amount of solvent used, and to meet the growing demand for natural bioactive compounds, some modern techniques are preferred.

#### 3.2.1. Ultrasound-Assisted Extraction (UAE)

Ultrasound-assisted extraction (UAE) has been identified as an effective and eco-friendly alternative to conventional extraction methods. This technique stands out for its simplicity, versatility, and cost-effectiveness due to the low volume of solvent required. It operates at mild temperatures, with less time and energy consumption, making it one of the most suitable extraction systems for large-scale operations [55,66,67]. For extraction purposes based on ultrasound, high-frequency waves (>2 MHz) are applied which favor the formation of cavitation bubbles. This phenomenon is responsible for the disruption of the cellular structure of the sample, facilitating the contact between solvent and cellular material, which consequently improves the mass transfer and increases the extraction efficiency [68,69]. In recent years, some authors applied ultrasound to extract phenolics from *Eucalyptus* biomass. Many of these studies focused on the optimization of several operational variables that affect the extraction efficiency of UAE, such as temperature, ultrasonication time, power, and frequency [42,55]. In this line, Bhuyan et al. [55] designed an optimization process for the recovery of phenolic compounds from *Eucalyptus robusta* leaves through RSM. The working parameters evaluated were temperature (30, 45, 60 °C), time (30, 60, 90 min), and power (150, 200, 250 W), using water as a solvent. The statistical results indicated that temperature was the variable with the greatest impact on the yield of TPC, followed by time and power. The proanthocyanidin content, as well as the antioxidant activity determined by ABTS, DPPH, and CUPRAC, was also mainly influenced by the temperature. On the other hand, the recovery of flavonoids was only affected by sonication time. Under optimized extraction conditions (250 W ultrasonic power for 90 min at 60 °C with water using a liquid-to-solid ratio (LSR) of 50 mL/g), UAE led to yield of TPC of 163.7 mg GAE/g, TFC of 6.2 mg RE/g, and proanthocyanidin of 6.1 mg CAE/g, with high antioxidant capacity (284.2, 302.9, and 680.6 mg TE/g for ABTS, DPPH and CUPRAC, respectively). In a previous study, Wong Paz et al. [57] also evaluated the effects of various operating parameters (% ethanol, time, and LSR) on the extraction of polyphenols from *E. camaldulensis* leaves. The authors indicated that the LSR had a strong positive impact on the TPC yield.

Recently, Gullón et al. [42] compared UAE (50 °C for 90 min) with conventional extraction (50 °C for 225 min) using 56% ethanol and an LSR of 10 mL/g for the isolation of antioxidants from *Eucalyptus* leaves. The results indicated that UAE led to similar values of TPC and TFC, but with lower energy consumption (0.082 vs. 0.177 kWh/g GAE), which confirms the suitability of UAE as a technology for the extraction of phytochemicals from natural sources.

#### 3.2.2. Microwave-Assisted Extraction (MAE)

Microwave-assisted extraction (MAE) is another promising alternative to recover phenolic compounds from *Eucalyptus* biomass due to its lower processing cost (requires less solvent, energy, and time), higher extraction yields, and better quality of the extracted compounds [19,70]. Microwave extraction is based on the application of electromagnetic radiation that causes an increase in temperature and pressure within the plant matrix, resulting in cell-structure disruption, which in turn facilitates the release of phytochemicals to the extractant [49,50]. As in UAE, various operational parameters including microwave power, LSR, temperature, and time are involved in the MAE process and must be carefully controlled to ensure high extraction performance and selectivity of recovered compounds. Some researchers also reported the optimization of extracted polyphenols from *Eucalyptus*. Bhuyan et al. [33] conducted a study to elucidate the optimal operating conditions of the microwave system to obtain maximum levels of phytochemicals, namely, phenolics, flavonoids, and proanthocyanidins with high antioxidant activity from *E. robusta* leaf. The independent variables studied were irradiation time (1–3 min), power (480–720 W), and LSR (12.5–50 mL/g) using water as a solvent. The authors noted that LSR had the greatest impact on the performance of TPC and proanthocyanidins, as well as on the antioxidant capacity determined by the ABTS assay. Under optimized extraction conditions (3 min, 600 W power, and an LSR of 50 mL/g), a TPC of 58.4 mg GAE/g and TFC of 19.2 mg RE/g were achieved. Furthermore, these conditions also led to an extract enriched in proanthocyanidins (6.2 mg CAE/g) and antioxidant activity of 74.9 mg TE/g for ABTS, 67.9 mg TE/g for DPPH, and 143.7 mg TE/g for CUPRAC, which represents between 62% and 67% of the maximum predicted by the model for these responses.

Fernández-Agulló et al. [58] compared MAE (65 °C, 10 min, LSR of 8.8 mL/g) with conventional extraction (50 °C, 90 min, LSR of 10 mL/g) for the recovery of bioactive compounds from *Eucalyptus* wood industrial wastes. According to the authors, maceration resulted in an extract with the best characteristics in terms of phenolic content and antioxidant potential; however, MAE was more efficient since it allowed significantly reducing the extraction time and the LSR. In a previous study, Gharekhani et al. [59] also compared MAE with UAE and traditional extraction. The results indicated that the MAE for 5 min extracted the same content of phenolic and flavonoid compounds as 60 min of UAE or conventional extraction at room temperature for 24 h. Recently, Gullón et al. [42] explored the use of MAE for the extraction of health-promoting phenolics from *Eucalyptus* leaves. The authors demonstrated that the use of microwaves allowed obtaining an extract with similar content of phenolic compounds and flavonoids, as well as antioxidant activity, but with an almost 14-fold lower energy consumption compared to conventional extraction.

#### 3.2.3. Supercritical Fluid Extraction (SFE)

Supercritical Fluid Extraction (SFE) has been established as an efficient green extraction technology used extensively to selectively isolate heat-sensitive high-value compounds from several natural sources. One important property of supercritical fluids is the possibility of modifying their solubility by changing their pressure and/or temperature; thus, these fluids can extract a wide spectrum of molecules of different polarities [48,71]. Of all possible supercritical fluids, carbon dioxide (CO_2_) is the most widely used because of its many advantages: nonflammable, noncorrosive, innocuous to human health, environmentally friendly, economical, abundant, and reusable. Furthermore, its critical parameters of low value (temperature of 31.1 °C and pressure of 73.8 bar) make it suitable for the extraction of thermolabile molecules [50,72]. Another important advantage of supercritical CO_2_ is that provides high-quality extracts free of organic solvents without the need for additional purification sequences [73]. However, due to the lack of polarity of CO_2_, it is ineffective to extract polar bioactive compounds (e.g., phenolics); hence, the addition of cosolvents (modifiers) such as ethanol or methanol is necessary to improve the extraction of these target compounds [74,75]. In general, the amount of cosolvent to be added varies from 1% to 15% [76].

Santos et al. [60] studied the influence of temperature, ethanol content, and CO_2_ flow rate on EY, TPC, the total amount of phenolic compounds quantified by HPLC, and antioxidant activity during SFE of biocompounds from *E. globulus* bark. The results indicated that the ethanol content had a positive impact on the four variables analyzed, whereas the temperature did not influence the phenolic profile, and the CO_2_ flow rate only affected the TPC.

In another study, Herzi et al. [61] compared SFE (90 bar, 40 °C, 30 min) with hydrodistillation (100 °C, 3 h) for the extraction of essential oil from two abundant species of the Tunisian forest, namely, *E. camaldulensis* and *E. cinerea*. The superiority of each technique was evaluated on the basis of performance, volatile chemical profile, phenolics, and antioxidant capacity. The results showed that SFE led to a higher extraction yield (27.5 vs. 23 g/kg for *E. cinerea* and 8.8 vs. 6.2 g/kg for *E. camaldulensis*) and extracts with stronger antioxidant activity (IC_50_ ABTS of 65 vs. 399 mg/L for *E. cinerea* and 128 vs. 183 mg/L for *E. camaldulensis*).

#### 3.2.4. Subcritical Water Extraction (SWE)

Subcritical water extraction (SWE) employs water at temperatures between 100 °C (boiling temperature) and 374 °C (critical temperature) at a pressure high enough to keep the water in a liquid state [77]. Under these conditions, the polarity of the water is lower; thus, the solubility of many organic compounds is improved compared to normal water [62] This process can be considered a green option to conventional extraction that uses organic solvents. In this context, Kulkarni et al. [62] evaluated the effectiveness of SWE for the recovery of antioxidants from *E. grandis* leaves. The authors compared SWE with solvent extraction in terms of extract performance and antioxidant potential. SWE led to similar extraction yields to conventional extraction (290 mg and 312 mg, respectively); however, SWE extracts presented 1.4-fold higher antioxidant capacity (measured by the ability of the samples to scavenge peroxynitrite free radicals in vitro) than the conventional extract (56.7% and 40.2%, respectively).

## 4. Biological Activities of Extracts Obtained from *Eucalyptus* Biomass

As mentioned previously, *Eucalyptus* biomass contains diverse phenolic compounds to which a plethora of therapeutic properties such as antibacterial, antifungal, antioxidant, neuroprotective, and anticancer activities, inter alia, are attributed.

### 4.1. Antioxidant Activity

The antioxidant activity of *Eucalyptus* biomass extracts is extensively reported in the literature. For example, Ashraf et al. [78] evaluated the antioxidant activity of different extracts (methanol, chloroform, and hexane) from *E. camaldulensis* leaves. Methanol extracts proved to have higher antioxidant potential than those obtained with the other solvents with IC_50_ values determined by free radical (DPPH) scavenging of 89.11 µg/mL (methanol), 154.8 µg/mL (chloroform), and 532.9 µg/mL (hexane). The authors indicated that the compounds responsible for this antioxidant activity were mainly phenolic acids (gallic acid, *p*-hydroxybenzoic acid, syringic acid, and vanillic acid) and flavonoids (catechin and quercetin). González et al. [51] studied the antioxidant capacity of ethanolic extracts obtained from the bark of two *Eucalyptus* species, namely, *E. globulus* and *E. nitens*. The results revealed that *E. nitens* bark extracts exhibited the highest antioxidant activity with DPPH values of 43.8 vs. 34.3 mg AAE/g dry bark.

In another study, Vuong et al. [52] investigated the antioxidant activity of an aqueous extract from *E. robusta* leaves through several in vitro methods, namely, ABTS, DPPH, hydrogen peroxide (H_2_O_2_), CUPRAC (cupric ion reducing antioxidant capacity), and FRAP (ferric reducing antioxidant power), and the results were compared with those obtained for α-tocopherol and ascorbic acid (considered as reference antioxidants). According to the authors, the extract presented an antioxidant capacity similar to that of ascorbic acid and significantly higher than that of α-tocopherol. They also highlighted that the potent antioxidant activity of these extracts could be enhanced with an additional purification step. The potential antioxidant effect of other polyphenolic extracts obtained from different *Eucalyptus* biomass is compiled in Table 3.

### 4.2. Antimicrobial Activity

In addition to the antioxidant properties associated with phenolic extracts derived from different biomass of the *Myrtaceae* family, several studies also reported their role as antimicrobial agents [14,37,41]. This is especially interesting because more and more microorganisms are becoming resistant to available antibiotics; thus, there is an urgent need to search for alternative biomolecules that can inhibit the broad spectrum of multidrug-resistant microorganisms [20,86]. Boulekbache-Makhlouf et al. [4] demonstrated the antimicrobial potential of crude extract from the fruit of *E. globulus* against three bacteria, namely, *Staphylococcus aureus*, *Bacillus subtilis*, and *Klebsiella pneumoniae*. The extract was effective against the two Gram-positive strains (*S. aureus*, *B. subtilis*) but had no inhibitory effect against *K. pneumoniae* (Gram-negative bacteria). Gullón et al. [41] also established that the ethanolic extract obtained from *Eucalyptus* leaf exhibited antibacterial activity against three Gram-positive (*S. aureus*, *Listeria innocua*, and *B. cereus*) and three Gram-negative bacteria (*Escherichia coli*, *Pseudomonas aeruginosa*, and *Salmonella* spp.). Although the authors did not attribute this activity to any specific compound, the evaluated extract contained several compounds, including β-eudesmol, γ-eudesmol, globulol, and *n*-hexadecanoic acid, with their combined action responsible for the antibacterial activity of the extract. In another interesting work, Gomes et al. [81] demonstrated that methanolic extracts of *Eucalyptus* leaves were effective against several *S. aureus* strains responsible for bovine mastitis. The authors related this positive effect to the combination of several phenolic compounds such as gallotannins, ellagic acid glycoside, and quercetin derivatives.

Some authors also confirmed the antifungal effect of phenolic components extracted from the bark of *E. globulus* and *E. nitens* [51]. These extracts displayed inhibition of the growth of the fungal species *Tinea versicolor*. Moreover, *E. globulus* extracts with a high concentration of volatiles (1,8-cineole, aromadendrene, α-pinene, and globulol) exhibited insecticidal activity against *Leptinotarsa decemlineata* larvae; therefore, these products could be suitable for the formulation of products intended to slow the growth of larvae in gardens and fields [2]. Overall, the results of these investigations open new opportunities for innovative therapeutic approaches using plant phenolics to reduce the drug resistance of many disease-causing microorganisms. Other studies on the antimicrobial potential of different extracts from *Eucalyptus* biomass are present in Table 3.

### 4.3. Other Activities

Some studies carried out in the last decade reported that *Eucalyptus* extracts are promising anticancer agents against different types of tumor cells [52,87,88,89]. For instance, Vuong et al. [52] evaluated anticancer activity of an aqueous extract from *E. robusta* leaves on various human cancer cell lines, namely, HT29 (colon), U87, SJ-G2, SMA (glioblastoma), MCF-7 (breast), A2780 (ovarian), H460 (lung), A431 (skin), Du145(prostate), BE2-C (neuroblastoma), and MiaPaCa-2 (pancreas). The data indicated that this extract displayed antitumor activity against all cancer cell lines tested. Furthermore, the authors also highlighted that *Eucalyptus* extract exerted a more significant cytotoxic effect on pancreatic cancer tumor cells compared to gemcitabine, which is considered one of the most relevant anticancer drugs for this type of cancer. Interestingly, the results also revealed that the compounds present in the *Eucalyptus* extract had few negative effects on normal pancreatic ductal epithelial cells. In previous work, Singab et al. [82] also demonstrated that the phenolic constituents of an aqueous acetone extract from *E. camaldulensis* Dehnh had a cytotoxic effect on tumor cell lines, particularly on MCF-7 (breast adenocarcinoma) and HCT-116 (colorectal adenocarcinoma) cell lines, in a dose-dependent manner.

Aqueous extracts from *E. globulus* Labill. also presented antitumor activity on colorectal, pancreatic, and non-small-cell lung cancer (HCT-15, PANC-1, and NCI-H460, respectively) [83]. In the particular case of NCI-H460, *E. globulus* decoction extract resulted in a dose-dependent decrease in the number of cells, limiting the cell cycle in the G0/G1 phase, with a reduction in cell proliferation and a rise in the expression of p53, p21, and cyclin D1 proteins.

The neuroprotective potential of *E. globulus* extracts was tested in vitro by González-Burgos et al. [79] in human neuroblastoma SH-SY5Y cells. The authors noted that these extracts have the ability ameliorate H_2_O_2_-induced oxidative stress damage through several mechanisms: decreasing the production of ROS and lipid peroxidation and increasing the cell viability, GSH (glutathione) concentration, and antioxidant enzyme activity.

The antiobesity role of *Eucalyptus* extracts was demonstrated by Iyyappan et al. [54] who observed a 90% inhibition of pancreatic lipase activity due to the phenolic compounds present in the extract. In another study, phenolic extracts from *E. grandis* × *E. urophylla* bark showed a potent inhibition of α-amylase and α-glucosidase activity associated with hyperglycemia.

## 5. Conclusions

On the basis of the information compiled in this review, we can conclude that the different parts of *Eucalyptus* biomass are excellent sources of value-added compounds with promising bioactivities. While it is true that, in the last few decades, the interest in the search for new compounds from natural sources with similar or enhanced properties as substitutes of the synthetic antioxidants or conventional antibiotics has increased, *Eucalyptus* species have been used in folk medicine to treat different ailments. Great efforts have been devoted to isolating and identifying the compounds present in the plants belonging to the Myrtaceae family, with phenols being mainly responsible for their beneficial properties. Moreover, the limitations of the conventional extraction technologies involve their operational time and temperature, harmful organic solvents, and degradation of the thermolabile compounds, which were overcome through the development and application of emerging technologies combined with greener smart solvents. These avant-garde techniques have allowed achieving high extraction yields with high antioxidant activities, while maintaining the properties of the obtained extracts. Until now, the in vitro biological activities of extracts from different *Eucalyptus* species were demonstrated, such as antioxidant, antimicrobial, anticarcinogenic, neuroprotective, and antihyperglycemic properties, among others. Therefore, the different parts of *Eucalyptus* species have great potential as natural sources to obtain active biologically compounds that respond to the increasing demand of consumers for natural products to treat some disorders and enhance their quality of life.

## Figures and Tables

**Table 1:** Phenolic compound characterization using analytical techniques reported for *Eucalyptus* extracts.

Specie	Source	Analytic Technique	Phenolic Compounds Detected	Reference
*E. globulus*	Leaves	HPLC	Rutin, quercitrin, chlorogenic acid, and ellagic acid	[31]
*E. globulus*	Leaves	HPLC–MS/MS	Chlorogenic acid, rutin, quercetin 3-glucuronide, and ellagic acid derivatives	[35]
*E. globulus*	Leaves	Py-GC/MS and FTIR	Possibly flavonoids and polyphenols	[41]
*E. maidenii*, *E. robusta*, E*. citriodora*, *E. tereticornis*, and *E. camaldulensis*	Leaves	GC/MS	Hydroquinone, hesperitin, naringenin, chlorogenic, catechin, and gallic acid, among others	[36]
Not specified	Commercial *Eucalyptus* leaf extract	HPLC and GC/MS	Gallic and ellagic acids, eucalyptone, and macrocarpals A–E	[34]
*E. globulus*	Leaves	HPLC–UV/MS	Hyperoside quercitrin myricetin, rutin, isoquercitrin, luteoline, apigenine, and quercetin, among others	[37]
*E. globulus*	Leaves	UHPLC–TOF-MS	Sideroxylonal A or B, quercetin 3-*O*-β-d-glucuronide, and ellagic acid– and methylellagic acid–pentoside, among others	[42]
*E.* *camaldulensis*	Leaves, buds, empty capsules, and seeds	GC/MS	Gallic acid, catechin, tricetin, hydroquinone, pyrogallol, hesperitin, and chlorogenic acid, among others	[38]
*E. globulus*	Bark	MALDI-TOF and RP-HPLC–ESI-TOF	Polygalloylglucoses mixtures (gallotannins), catechin, epicatechin, and ellagic acid, among others	[39]
*E. grandis, E. maidenii and E. urograndis* (*E. grandis × E. urophylla*)	Bark	HPLC-UV, HPLC–MS/MS, and MS^n^	Ellagic acid–rhamnoside, dihydroxy-isopropylchromone–hexoside, dihydroxy-(methylpropyl) isopropylchromone–hexoside, epicatechin, quercetin–glucuronide, catechin, and chlorogenic acid, among others	[15]
By-products from the *Eucalyptus* wood industry	Screw water, condensates, and concentrate	GC/MS and LC–MS/MS	Gallic acid, protocatechuic acid, chlorogenic acid, 3,4-dihydroxybenzaldehyde, 4-hydroxybenzaldehyde, geranyl acetate, geranyl butyrate, *trans*-geraniol, sesquiterpenes (alloaromadendrene, ledene, α-Selinene, β-cadinene)	[40]
*E. globulus*	Wood industrial wastes	RP-HPLC–ESI-TOF	Ellagic acid, myricetin 3-*O*-rhamnoside, and quercetin 3-glucoside	[43]
*E. globulus*	Leaves	RP-HPLC and ^13^C- and ^1^H-NMR	Cypellocarpin A, eucaglobulin, cuniloside, and (1*S*, 2*S*, 4*R*)-*trans*-2-hydroxy-1,8-cineole β-d-glucopyranoside	[16]

HPLC: high-performance liquid chromatography; GC/MS: gas chromatography–mass spectrometry; MALDI-TOF: matrix-assisted laser desorption ionization time-of-flight; RP-HPLC–ESI-TOF: reverse-phase high-performance liquid chromatography electrospray ionization time-of-flight; Py-GC/MS: pyrolysis and gas chromatography coupled with mass spectrometry; UHPLC–TOF-MS: ultra-high-performance liquid chromatography coupled with trapped ion mobility spectrometry and TOF high-resolution mass spectrometry; FTIR: Fourier-transform infrared spectroscopy; NMR: nuclear magnetic resonance.

**Table 2:** Extraction technologies for obtaining phenolic compounds from Eucalyptus biomass.

Source	Extraction Conditions	Yield	Phenolic Compounds and Antioxidant Activities	Reference
**Conventional Extraction**				
*E. globulus* leaves	50 °C, 225 min, 56% ethanol, using an LSR of 20 mL/g in an orbital shaker at 120 rpm	32.7%	TPC: 92.7 mg GAE/g dw, TFC: 53.7 mg RE/g dw, DPPH: 205.4 TE/g dw, ABTS: 363.4 TE/g dw and FRAP: 185.2 mg TE/g dw	[41]
*E. globulus* and *E. nitens* bark	51% methanol, using an LSR of 60 mL/g for both species and at 52.85 °C for *E. nitens* and 45.85 °C for *E. globulus*	Not specified	*E. nitens:* TPC: 48.0 mg GAE/g dry bark and DPPH: 43.8 mg AAE/g dry bark, *E. globulus*: TPC: 37.8 mg GAE/g dry bark and DPPH: 34.3 mg AAE/g dry bark	[51]
*E. robusta* leaves	85 °C, 15 min, and an LSR of 20 mL/g	Not specified	TPC: 124.9 mg GAE/g	[52]
*E. globulus* stump wood	*n*-hexane, ethanol, methanol, and 75% aqueous ethanol	Methanol: 12.3%, ethanol: 9.3%, 75% ethanol: 8.1%, and hexane: 2.7%	TPC: (mg GAE/g extract): 460, 451.1, 444.6 and 25.9 for ethanol, methanol, 75% ethanol and n-hexane, respectively, TFC: (mg QE/g extract): 33.6, 43.1, 44.9, and 47.2 for ethanol, methanol, 75% ethanol, and *n*-hexane, respectively, IC_50_ value by DPPH assay (mg/mL): 5.9, 6, 6.4, and 189.9 for ethanol, methanol, 75% ethanol, and *n*-hexane, respectively	[53]
*E. globulus* bark	100 °C, 1.5% of Na_2_SO_3_ without NaOH	Not specified	TPC: 21.9 g GAE/100 g extract, FRAP: 132.8 nmol AAE/100 g extract	[39]
*E. globulus* leaves	Methanol, ethanol, and chloroform (100% each) using an LSR of 10 mL/g in a shaking water bath at 150 rpm for 2 days at room temperature	Not specified	The highest phenolic content (mg TAE/100 g dw) was obtained in extracts of methanol (8.8) followed by ethanol (7.9) and chloroform (4.6)	[54]
*E. camaldulensis* leaves	Ethanol, methanol, acetone, ethyl acetate (95%, 70%, and 30%), and distilled water using an LSR of 10 mL/g for 72 h	70% acetone: 46.6 mg/g dw, 30% methanol: 38.2 mg/g dw and 95% methanol: 34.6 mg/g dw	Acetone extracts exhibited the best antioxidant activity: 57.6, 50.5, and 35.5 mg/g dry weight for 95%, 70%, and 30%, respectively	[38]
*E. grandis*, *E. urograndis* and *E. maidenii* bark	50% methanol at room temperature for 24 h under constant stirring using an LSR of 100 (v/m)	*E. grandis*: 10.5%, *E. urograndis*: 15.2%, *E. maidenii:* 13.2%	*E. grandis*: TPC: 40.6 mg GAE/g of bark, *E. urograndis*: TPC: 56.9 mg GAE/g of bark, *E. maidenii:* TPC: 26.9 mg GAE/g of bark	[15]
*E. globulus* leaves	Chloroform, ethanol, methanol, or 70% methanol at room temperature for 8 h using an LSR of 12.5 mL/g in an orbital shaker	Not specified	TPC: 0.19, 1.9, 8.1, and 12.9 mg GAE/g dw, for chloroform, ethanol, methanol, and 70% methanol	[35]
*E. globulus* leaves	ChEG, ChX, ChG, and GCA using an LSR of 10 mL/g at 50 °C for 60 min	Not specified	TPC: 69.9 mg GAE/g dw, TFC: 45.4 mg RE/g dw, DPPH: 68 mg TE/g dw, ABTS: 89.9 mg TE/g dw, FRAP: 66.3 mg TE/g dw	[42]
**Ultrasound-Assisted Extraction (UAE)**				
*E. robusta* leaves	250 W ultrasonic power for 90 min at 60 °C using water and an LSR of 50 mL/g	Not specified	TPC: 163.7 mg GAE/g, TFC: 6.2 mg RE/g, proanthocyanidins: 6.1 mg CAE/g, ABTS: 284.2 mg TE/g, DPPH: 302.9 mg TE/g, CUPRAC: 680.6 mg TE/g	[55]
*E. sideroxylon* bark	50% of ethanol for 60 min at 50 °C using an LSR of 10 (v/m)	50.0	TPC: 440.7 mg GAE/g of extract, TFC: 204.4 mg CAE/g of extract, Tannins: 395.0 mg CAE/g of extract, DPPH: 648.8 mg Trolox/g of extract, FRAP: 5247 mM Fe^2+^/g of extract	[56]
*E. globulus leaves*	56% of ethanol for 90 min at 50 °C using an LSR of 10 mL/g	Not specified	TPC: 84 mg GAE/g dw, TFC: 47.2 mg RE/g dw, DPPH: 156.6 TE/g dw, ABTS: 241.1 TE/g dw and FRAP: 84.7 mg TE/g dw	[42]
*E. camaldulensis* leaves	35% ethanol for 46.8 min using an LSR of 12 mL/g	Not specified	TPC: 13.9 mg GAE/g of dry plant material	[57]
**Microwave-Assisted Extraction (MAE)**				
*E. robusta* leaf	3 min, 600 W power, and an LSR of 50 mL/g using water as a solvent	Not specified	TPC: 58.4 mg GAE/, TFC: 19.2 mg RE/g, proanthocyanidins: 6.2 mg CAE/g, ABTS: 74.9 mg TE/g, DPPH: 67.9 mg TE/g CUPRAC: 143.7 mg TE/g	[33]
*E. globulus* leaves	56% ethanol for 7 min using an LSR of 10 mL/g	Not specified	TPC: 79.4 mg GAE/g dw, TFC: 39.4 mg RE/g dw, DPPH: 141.2 TE/g dw, ABTS: 187.4 TE/g dw and FRAP: 105.8 mg TE/g dw	[42]
*E. globulus* wood industrial wastes	Ethanol for 10 min at 65 °C using an LSR of 8.8 mL/g	2.3%	TPC: 65.1 g GAE/100 g extract, FRAP: 5458 nmol AAE/mg extract	[58]
*E. camaldulensis* Dehn leaves	50% ethanol, 600 W power for 5 min using an LSR of 20 mL/g	Not specified	TPC: 76.6 mg GAE/g sample, TFC: 5.8 mg QE/g sample	[59]
**Supercritical Fluid Extraction (SFE)**				
*E. globulus* Labill bark	70 °C, 20% ethanol as cosolvent, CO_2_ flow rate of 10 g/min at 300 bar	0.5%	TPC: 57.2 mg GAE/g of extract, PC-HPLC: 119.5 mg/g of extract, and DPPH: 49.7 mg AAE/g of extract	[60]
*E. camaldulensus* and *E. cinerea* leaves	SFE: 40 °C, for 30 min and 90 bar Hydrodistillation (HD): 100 °C for 3 h	*E. camaldulensus*: 8.8 g/kg for SFE and 6.2 g/kg for HD, *E. cinerea*: 27.5 g/kg for SFE and 23 g/kg for HD	The extracts obtained by SFE exhibited a powerful antioxidant activity compared to those obtained by HD	[61]
**Subcritical Water Extraction (SWE)**				
*E. grandis* leaves	SWE: 160 °C at 3 MPa, SE: 7 days with constant stirring using methanol as solvent	SWE: 290 mg, SE: 312 mg	AA for SWE of 56.7% and for SE of 40.2%	[62]

Ch: choline chloride; EG: ethylene glycol; X: xylitol; CA: citric acid; LSR: liquid-to-solid ratio; TAE: tannic acid equivalent; CAE: catechin equivalents; GAE: gallic acid equivalents; RE: rutin equivalents; EY: extraction yield; TPC: total phenolic content; TFC: total flavonoid content; DPPH: α,α-diphenyl-β-picrylhydrazyl radical scavenging; ABTS: 2,2’-azino-bis-3-ethylbenzothiazoline-6-sulfonic acid; FRAP: ferric reducing antioxidant power; CUPRAC: cupric reducing antioxidant capacity; AAE: ascorbic acid equivalent; QE: quercetin equivalent; PC-HPLC: phenolic compounds quantified by HPLC; SE: solvent extraction; HD: hydrodistillation; SFE: supercritical fluid extraction; SWE: subcritical water extraction; AA: antioxidant activity; DW: dry weight.

**Table 3:** Biological activities of the extracts obtained from *Eucalyptus* biomass.

Source/Type of Extract	Outcomes	Reference
**Antioxidant Activity**		
Leaves of *E. camaldulensis* Dehn. extracted with three different solvents (methanol, chloroform, and hexane)	IC_50_ (µg/mL) determined by DPPH: 89.1 µg/mL (methanol), 154.8 µg/mL (chloroform), 532.9 µg/mL (hexane)	[78]
Fruits of *E. globulus* extracted with 70% acetone–water containing 0.5% acetic acid	High reducing power and moderate inhibition of lipid peroxidation of linoleic acid emulsion; reducing power: IC_50_ = 39.5 µg/mL, lipid peroxidation inhibition = 51.3%	[4]
*E. globulus* leaves extracted with 70% ethanol	DPPH: 15.3 μg QE/mg plant material, ABTS: 9.0 μg TE/mg plant material, HAPX: 61.2%	[37]
Ethanolic extracts obtained from the bark of *E. globulus* and *E. nitens*	DPPH (mg AAE/g dry bark): 43.1 for *E. nitens* and 35 for *E. globulus*	[51]
Extracts of *E. globulus* leaves (acetone, methanol, and ethanol as solvents)	Acetone extracts presented the highest antioxidant activity using ABTS and CUPRAC (10.1 and 3.7 mmol/g respectively); the highest value for DPPH was seen for methanol extract (1.6 mmol/g), and ethanol extracts led to the highest values for FRAP and TFPH assays (9.8 and 1.8 mmol/g, respectively).	[79]
Stumps of *E. globulus* using *n*-hexane, methanol, ethanol, and 75% ethanol	IC_50_ (mg/L) determined by DPPH: *n*-hexane 170.3–369.3 mg/L, ethanol 5.9–11.3 mg/L, methanol 6–12.5 mg/L, and 75% ethanol 6.35–17.3 mg/L	[53]
Bark of *E. sideroxylon*	DPPH: 648.8 mg Trolox/g of extract, and FRAP: 5247 mM Fe^2+^/g of extract	[56]
Methanolic extracts of *E. grandis* wood from Portugal, Brazil, and South Africa	IC_50_ determined by DPPH: *E. grandis* Portugal: 6. 2 µg/mL, *E. grandis* Brazil: 5.1 µg/mL, *E. grandis* South Africa: 6.1 µg/mL. ABTS: *E. grandis* Portugal: 10.0 mg AAE/g of dry wood, *E. grandis* Brazil: 23.4 mg AAE/g of dry wood, *E. grandis* South Africa: 13.9 mg AAE/g of dry wood	[80]
*E. robusta* leaf aqueous extract	ABTS: 832.8 mg BHT/g, DPPH: 1403.9 mg BHT/g, hydrogen peroxide (H_2_O_2_): 1447.5 mg BHT/g, CUPRAC: 715.7 mg BHT/g, FRAP: 1638.2 mg BHT/g	[52]
**Antimicrobial Activity**		
Fruits of *E. globulus* extracted with 70% acetone–water containing 0.5% acetic acid	Growth inhibition of *Bacillus subtilis* (MIC of 30 µg/mL) and *Staphylococcus aureus* (MIC of 80 µg/mL). No inhibition of *Klebsiella pneumoniae*	[4]
Ethanolic extract from *E. globulus* leaves	MIC values (mg/mL): *S. aureus* (35), *Listeria innocua* (30), *B. cereus* (40), *Escherichia coli* (40), *Pseudomonas aeruginosa* (45), and *Salmonella* spp. (45) MBC values (mg/mL): *S. aureus* (40), *L. innocua* (35), *B. cereus* (45), *E. coli* (50), *P. aeruginosa* (50), and *Salmonella* spp. (50)	[41]
Ethanolic extract from *E. globulus* leaves	MIC values (µg/mL): *S. aureus* 50, *B. subtilis* > 100, *L. monocytogenes* 30, *E. coli* > 100, *S. typhimurium* > 100	[37]
Methanolic extract from *E. globulus* Labill. leaves	MIC values varied between 0.19 and 0.39 mg/mL depending on the *S. aureus* strain tested	[81]
Phenolic components from the bark of *E. globulus* and *E. nitens*	MIC values (μg/μL): 7.5 for *E. globulus* and 15 for *E. nitens*	[51]
Extract from *E. globulus* leaves	High acute toxicity with LD_50_ = 38 µg against *Leptinotarsa decemlineata* larvae	[2]
Extracts of *E. globulus* (stump wood, stump bark, and industrial chips)	The different extracts exhibited low MIC values (0.156–10 mg/mL) against several strains of *S. aureus* (including MRSA strains) food-borne pathogens (*B. cereus* and *L. monocytogenes*), *Candida* strains, and some Gram-negative bacteria (*E. coli*, *P. aeruginosa*, *K. pneumoniae*)	[53]
**Other Activities**		
Aqueous extracts from *E. robusta* leaves	Antitumor activity against cancers of colon, glioblastoma, breast, ovarian, lung, skin, prostate, neuroblastoma, and pancreas	[52]
Aqueous acetone leaf extract of *E. camaldulensis* Dehnh	Cytotoxic effect on tumor cell lines: breast adenocarcinoma, human epithelial laryngeal carcinoma, hepatocellular carcinoma, human cervix adenocarcinoma, colorectal adenocarcinoma, and Caco-2 colon adenocarcinoma	[82]
Aqueous extracts from *E. globulus* Labill.	Antitumor activity on colorectal, pancreatic, and non-small-cell lung cancer	[83]
Isolated compounds of *E. globulus* leaves: 2, 2, 8-trimethyl-6-formyl-chrom-3-ene 7-*O*-β-d-glucopyranoside, quercetin 3-*O*-α-l-4C1 arabinopyranoside-2″-gallate, cornusiin B, and eucalbanin B	Nephroprotective role against diabetes mellitus and kidney stone disease	[84]
*E. globulus* leaves	Neuroprotective activity	[79]
*E. globulus* leaves	Antiobesity activity: 90% inhibition of pancreatic lipase activity	[54]
*E. grandis* × *E. urophylla* bark	Prevention of hyperglycemia through the inhibition of α-glucosidase and α-amylase activities	[85]

QE: quercetin; TE: Trolox equivalent; HAPX: hemoglobin/ascorbate peroxidase activity inhibition assay; AAE: ascorbic acid equivalent; BHT: butylated hydroxytoluene; MIC: minimum inhibition concentration; MBC: minimum bactericidal concentration.
